# Understanding implementation costs of a pediatric weight management intervention: an economic evaluation protocol

**DOI:** 10.1186/s43058-022-00287-1

**Published:** 2022-04-05

**Authors:** Tzeyu L. Michaud, Jennie L. Hill, Kate A. Heelan, R. T. Bartee, Bryce M. Abbey, Ali Malmkar, John Masker, Caitlin Golden, Gwenndolyn Porter, Russell E. Glasgow, Paul A. Estabrooks

**Affiliations:** 1 Department of Health Promotion, College of Public Health, University of Nebraska Medical Center, Omaha, NE 68198 USA; 2 Center for Reducing Health Disparities, College of Public Health, University of Nebraska Medical Center, Omaha, NE USA; 3 Department of Population Health Sciences, University of Utah, Salt Lake City, UT USA; 4 Kinesiology and Sport Sciences Department, University of Nebraska at Kearney, Kearney, NE USA; 5 Department of Family Medicine and Adult and Child Outcomes and Delivery Sciences, University of Colorado Anschutz Medical Campus, Aurora, CO USA; 6 Department of Health and Kinesiology, College of Health, University of Utah, Salt Lake City, UT USA

**Keywords:** Childhood obesity, Implementation strategy, Dissemination, RE-AIM, Activity-based costing

## Abstract

**Background:**

Understanding the cost and/or cost-effectiveness of implementation strategies is crucial for organizations to make informed decisions about the resources needed to implement and sustain evidence-based interventions (EBIs). This economic evaluation protocol describes the methods and processes that will be used to assess costs and cost-effectiveness across implementation strategies used to improve the reach, adoption, implementation, and organizational maintenance of an evidence-based pediatric weight management intervention- Building Health Families (BHF).

**Methods:**

A within-trial cost and cost-effectiveness analysis (CEA) will be completed as part of a hybrid type III effectiveness-implementation trial (HEI) designed to examine the impact of an action Learning Collaborative (LC) strategy consisting of network weaving, consultee-centered training, goal-setting and feedback, and sustainability action planning to improve the adoption, implementation, organizational maintenance, and program reach of BHF in micropolitan and surrounding rural communities in the USA, over a 12-month period. We discuss key features of implementation strategy components and the associated cost collection and outcome measures and present brief examples on what will be included in the CEA for each discrete implementation strategy and how the results will be interpreted. The cost data will be collected by identifying implementation activities associated with each strategy and using a digital-based time tracking tool to capture the time associated with each activity. Costs will be assessed relative to the BHF program implementation and the multicomponent implementation strategy, included within and external to a LC designed to improve reach, effectiveness, adoption, implementation, and maintenance (RE-AIM) of BHF. The CEA results will be reported by RE-AIM outcomes, using the average cost-effectiveness ratio or incremental cost-effectiveness ratio. All the CEAs will be performed from the community perspective.

**Discussion:**

The proposed costing approach and economic evaluation framework for dissemination and implementation strategies and EBI implementation will contribute to the evolving but still scant literature on economic evaluation of implementation and strategies used and facilitate the comparative economic analysis.

**Trial registration:**

ClinicalTrials.gov NCT04719442. Registered on January 22, 2021.

**Supplementary Information:**

The online version contains supplementary material available at 10.1186/s43058-022-00287-1.

Contributions to the literature

- We report a novel framework to assess costs and cost-effectiveness across implementation strategies used to improve the RE-AIM outcomes of an evidence-based pediatric weight management intervention
- We identify and present the strategy costs across RE-AIM dimensions to clarify sometimes fuzzy boundaries between these strategies
- We provide a pragmatic and replicable methodology for costing dissemination and implementation (D&I) strategies to help stakeholders or community organizations decide what strategies are most efficient at achieving the goal of adoption, implementation, and sustainability and demonstrate the usability of the proposed time-tracking tool to facilitate cost assessments of D&I strategies in the community settings

## Background

Childhood obesity is associated with increased risk for a variety of cardiovascular and other diseases and has been documented to predict adult obesity which results in personal health challenges and imposes an economic burden to the healthcare system [1, 2]. Although pediatric weight management interventions (PWMIs) or prevention programs are becoming prevalent, families from micropolitan (i.e., cities <50,000 populations) and surrounding rural areas have limited access to PWMIs that are typically offered in larger urban areas. Expanding the availability of evidence-based PWMIs and increasing capacity in micropolitan regions to implement these interventions can have a significant public health impact. Systems-based approaches that engage multiple community-organizational partners to share implementation responsibility have been proposed as a potential pathway to increasing PWMI access in micropolitan communities [3].

Integrated system-based approaches typically include horizontal components (i.e., engagement of organizations across a community) and vertical components (i.e., engagement of administrative decision makers and staff that would ultimately implement the PWMI) [4]. Both horizontal and vertical system components provide opportunities for applying dissemination and implementation (D&I) strategies that may improve the likelihood that evidence-based PWMIs can be adapted, adopted, implemented, and sustained in micropolitan areas [3, 5]. For example, a fund and contract strategy, involving research funding announcement, application review, and contractual agreements for implementation, can be used to improve the adoption of PWMIs in communities with the readiness for implementation [6]. Moreover, the use of an action learning collaborative (LC) strategy that encompasses a variety of activities to support communities in improving PWMI reach, effectiveness, adoption, implementation, and maintenance (RE-AIM [7]) (e.g., structured learning/training sessions, goal-setting and feedback, sustainability action planning) can be used to increase community engagement across organizations and facilitate capacity building for PWMI implementation [8, 9].

Despite the proposed promise of these approaches, it is necessary to understand both the ability of these strategies to improve RE-AIM outcomes as well as the costs and resources needed to execute D&I strategies [10–12]. Unfortunately, study findings from those that have conducted economic evaluations of D&I strategies are mixed and thus the economic feasibility of D&I strategy use remain unclear. In studies that have applied economic analysis of LCs [13, 14], a cost-effectiveness analysis (CEA) focusing on pre-post changes in clinician’s competency on trauma-focused cognitive behavioral therapy and youth’s trauma-related mental health found the LC did not produce a favorable cost-effectiveness ratio with clinician competency as the outcomes measure. However, the subsequent reductions in youth psychopathology demonstrated a high cost-effectiveness. More broadly, two recent review studies [15, 16] identified articles examining the costs, consequences, and cost-effectiveness of strategies designed to influence the adoption of public or population-level interventions in community settings. Both reviews shared the methodology limitation that the number of studies that they identified and synthesized, especially among studies associated with the implementation or implementation strategies, and a plethora of terms and definitions of D&I strategies making cross-study comparisons related to CEA challenging. Moreover, no detailed discussion of costing methods was found in their reviews.

As a result of the challenge of summarizing CEAs relative to D&I strategies, there is a movement to increase the specification of both D&I strategies and the resulting economic evaluation [17]. The recent effort made by the “Economics and Cost” action group of the Consortium for Cancer Implementation Science is an example of this movement. In a series of papers [10–12, 18], this effort provided common definitions, costing methods, developing costing guidance, and guidance to build collaborations across disciplines [19]. Complementary to the collection of articles, this paper provides a description of an economic evaluation across various D&I outcomes using RE-AIM [7] and methods to cost D&I strategies mapped to specific outcomes related to PWMI implementation in micropolitan settings. Specifically, it outlines the methods and processes of the prospective within-trial economic evaluation of D&I strategies to be undertaken within a hybrid type III effectiveness-implementation (HEI) trial to improve RE-AIM outcomes of evidence-based PWMIs in micropolitan communities. We will examine costs and cost-effectiveness of D&I strategies focused on enhancing PWMI (1) reach (i.e., the number, proportion, and representativeness of program participants relative to the intended audience), (2) adoption (i.e., the number and proportion of communities that agree to deliver the PWMI), (3) implementation (i.e., adherence to program protocol), (4) organizational maintenance (i.e., sustained implementation to addition cohorts of families over time), and (5) their overall impact on the effectiveness (i.e., reduction in BMI z scores). The cost-effectiveness results will provide data for payers, policymakers, and providers to make informed decisions about specific strategies for specific RE-AIM outcome targets, especially in organizations or communities where resources are scarce.

## Methods

### Study design

This economic evaluation protocol is embedded within a type III HEI trial, aiming to test multilevel D&I strategies that focus on the RE-AIM outcomes of an evidence-based PWMI (i.e., Building Health Families [BHF] [20–22]). Details of the overarching trial have been published elsewhere [3, 5, 23] and information on that trial is presented here as it relates to the economic evaluation of strategies that target RE-AIM outcomes (see Table 1). Specifically, the trial includes three primary D&I strategies—fund and contract, an online, packaged implementation blueprint, and an action LC. In addition, the implementation blueprint includes activities focused on training, program implementation, and referral resources to improve program reach. Similarly, the LC includes activities focused on network weaving, used to promote information sharing within and outside the organizations building upon existing working relationships [6], consultee-centered training, and sustainability action planning. The study is intended to (1) identify local demand for BHF, a PWMI that was used as the basis for the online, packaged implementation blueprint (referred to hereafter as the BHF Online Training Resources and Program Package [5]); (2) examine potential determinants of BHF adoption; and (3) compare BHF RE-AIM outcomes in communities that participate in the LC to those that do not. As a pilot type III HEI, the primary aim is to determine if the LC communities document better D&I outcomes (reach, adoption, implementation, BHF sustainability) and the secondary aim is to determine the relative effectiveness of BHF between communities that participate in the LC versus those that do not. Of note, regardless of LC participation status, communities are exposed to strategies of fund and contract and BHF Online Training Resources and Program Package. The trial protocol is registered with clinicaltrials.gov (NCT04719442).

**Table 1:** Cost and outcome measures for the within-trial economic evaluation by the RE-AIM outcomes

Outcomes	Corresponding strategy	Cost of BHF-LC (C_**LC**_)	Cost of BHF-PO (C_**PO**_)	Outcome measure (E)	ACER/ICER	Interpretation
**R**each	-LC- consultee-centered training focusing on recruitment at schools and clinics -Develop a packaged implementation blueprint- referral process	-LC training and consultation sessions -BHF recruitment training -Recruitment activities	-BHF recruitment training -Recruitment activities	-Number, proportion, & representativeness of eligible families screened	C_LC_-C_PO_/E_LC_-E_PO_	Cost of per additional person recruited
**E**ffectiveness	-BHF program training and delivery	-All LC activities -BHF program training and delivery	-BHF program training and delivery	-Reduction in BMI *z*-scores	C_LC_-C_PO_/E_LC_-E_PO_	Cost per additional BMI *z*-score reduced
**A**doption	-Fund and contract	-Response to RFA from potential community stakeholders and organizations	-Response to RFA from potential community stakeholders and organizations	-Objective count of communities responding to RFA and contracted to implement BHF	C_all_/E_all_	Cost per community adoption
	-LC- consultee-centered training with focus on BHF cohort adoption -Develop a packaged implementation blueprint- BHF online training resources & package	-LC training and consultation sessions -Online training and program packaging -BHF program training and delivery	-Online training and program packaging -BHF program training and delivery	-Objective count of # of BHF cohorts initiated	C_LC_-C_PO_/E_LC_-E_PO_	Cost per additional cohort initiated
**I**mplementation	-LC- consultee-centered training with focus on implementation fidelity	-LC training and consultation sessions	None	-Average fidelity scores from the direct observation	C_LC_/E_LC_-E_PO_	Cost per additional changes in fidelity scores
	-Develop a packaged implementation blueprint- BHF online training resources & package	-Online training and program packaging	-Online training and program packaging	-Average knowledge check scores from BHF online training resources -Summarized implementation fidelity assessed via direct observation.	C_all_/E_all_	Cost per knowledge score/unit of implementation fidelity
**M**aintenance- setting level	-LC- sustainability action planning	-LC training and consultation sessions	None	-Objective count of BHF cohort continued	C_LC_/E_LC_-E_PO_	Cost per additional 3rd cohort initiated

*RE-AIM* Reach, effectiveness, adoption, implementation, and maintenance, *BHF* Building Healthy Families, *LC* Learning collaborative, *PO* Package only, *RFA* Request for application, *ACER* Average cost-effectiveness ratio, *ICER* Incremental cost-effectiveness ratio, *LOI* Letter of intent

Each of the D&I strategies is described below. To ensure clear communication and transparency, we used Proctor and colleagues’ framework [24] to specify implementation strategies. The specification of each D&I strategy by the *actor* (who enacts the strategy?), the *action*(s) (what are the specific actions, steps, or processes that need to be enacted?), the *action target* (what constructs are targeted? What is the unit of analysis?), *temporality* (when is the strategy used?), *dose* (what is the intensity?), and implementation *outcome* (what implementation outcome(s) are likely to be affected by each strategy?) is presented in Table 2. We also individually assess the costs of each strategy.

**Table 2:** Labor activity specification of dissemination and implementation strategies used in the HEI trial by the Proctor framework [24]

Strategy [3]	Outcome^**a**^	Action	Actor	Action target	Temporality	Dose (frequency)	Dose (duration)^**c**^
**Develop packaged implementation blueprint**^**b**^ • BHF Online Training Resources and Program Package • Referral process	**Reach** -Number, proportion, & representativeness of eligible family screened	-Training activities on developing referral channels across media, school, and clinical sources.	-Electronic resource and research team	BHF-LC & BHF-PO	Early	One module that can be repeated	As needed
	**Adoption** -Objective count of the number of BHF cohorts initiated	-Training material modification -Program resource provision	-Electronic resource and Research team -Trifoia	BHF-LC & BHF-PO	Early	18 training modules	Duration of each programming & trouble shoot
	**Implementation** -Observed adherence to protocol and increased facilitator capacity	-Monitor training progress and the completion of knowledge & fidelity checklists	-Project coordinator	BHF-LC & BHF-PO	Early-mid	18 training modules	Duration of each training session
**Fund and contract** • Network weaving (recruitment of community) • Obtain formal commitment	**Adoption** -Objective count of communities & organizations responding to call for letter of intent -Objective count of communities & organizations responding RFA and contracted to implement BHF	-Draft the LOI -Draft the full application -Review the application materials -Meetings to discuss the applications & make a decision -Contact with the awardees -Obtaining the written memorandums	-Research team & CAB -Research team & CAB -Research team -Research team & CAB -Project coordinator -Project coordinator	Interested communities	Early	One-time for interested community	Duration of the meetings/telecommunications
**Learning Collaborative**^**b**^ • Network weaving (recruitment of BHF participants) • Consultee-centered training • Sustainability action planning	**Reach** -Number, proportion, & representativeness of eligible family screened -Representativeness of participating low-income families	-Document screening & engagement of low-income families -Create learning session materials that focus on identifying recruitment sources and channels to improve reach—with an emphasis on schools and clinics. -Delivery learning sessions that provide opportunities for community implementation teams to action plan recruitment procedures -Provide individual/group consultation on recruitment methods and outcomes -Follow up on action plans and outcomes using lighting report protocol -Provide action period consultation and reporting opportunities	-Research team -Research team -Research team -Research team -Research team -Research team	BHF-LC	Early-mid	Frequency of the sessions focusing on reach	Duration of the sessions
	**Adoption** -Objective count of the number of BHF cohorts initiated	-Create learning session materials -Delivery learning sessions -Provide individual/group consultation -Conduct knowledge/fidelity checklist -Provide feedback at each learning session -Complete pragmatic evaluations	-Research team -Research team -Research team -Research team -Research team -Research team	BHF-LC	Mid-late	Frequency of the training sessions focusing on adoption	Duration of the sessions
	**Implementation** -Observed adherence to protocol and family engagement at each BHF session -Instructor self-reported fidelity at each BHF session	-Create learning session materials -Delivery learning sessions -Provide individual/group consultation -Conduct knowledge/fidelity checklist -Provide feedback at each learning session -Complete pragmatic evaluations	-Research team -Research team -Research team -Research team -Research team -Research team	BHF-LC	Mid-late	Frequency of the training sessions focusing on implementation	Duration of the sessions
	**Maintenance** -Survey/checklist at 12 months -Objective count of sustained implementation of cohorts in the year following completion of the LC intervention	-Develop sustainability needs assessment checklists -Develop funding matrix development tools -Develop key partner identification and engagement -Develop an ongoing assessment & feedback -Conduct sustainability needs assessment -Examine current and future resources, payer and payment models -Integrate into regular learning meetings & action periods	-Research team & CAB -Research team -Research team -Research team & CAB -Research team -Research team & CAB -Research team	BHF-LC	Late	Frequency of activities/events focusing on maintenance	Duration of the activities

*BHF* Building Healthy Families, *LC* learning collaborative, *PO* Package only, *CAB* community advisory board, *LOI* Letter of intent

^a^Costs are divided among RE-AIM (reach, effectiveness, adoption, implementation, and maintenance) outcomes and are estimated with actions/activities specified for that specific outcome

^b^Costs are divided among implementation outcomes associated with the strategy, based on the activities specified in the action column

^c^Preparation time and travel time (if any) and post-session follow-up (e.g., summary note) are also included

### Fund and contract dissemination strategy focused on adoption

Fund and contract dissemination strategies typically include a request for applications (RFA) to deliver an evidence-based intervention, contracting to ensure understanding of what delivery will entail, and some sort of funding to improve local motivation to adopt the given intervention [6]. As part of this trial, the fund and contract dissemination strategy is used to identify communities with strong cross-organization partnerships and a priority to address childhood obesity locally. Table 2 includes a full specification of this strategy and the HEI trial includes 2 phases—a call for letters of intent (LOI) and an invitation for full proposals based on LOI review. Both processes are developed by the research team and reviewed and revised in partnership with the community advisory board (BHF-CAB). The call for LOIs focuses on describing the attributes of the BHF Online Training Resources and Program Package (i.e., relative advantage, compatibility, complexity, observability, and trialability [25]). The call also highlights financial resources available for the community and the need for a horizontal (i.e., across community organizations) and vertical (i.e., decision makers and doers) systems-based approach to BHF implementation. The target of the fund and contract strategy was to increase community adoption of BHF [23].

### Developing packaged implementation blueprint: BHF Online Training Resources and Program Package focused on adoption and implementation fidelity

A detailed description of the BHF Online Training Resources and Program Package has been published previously [5]. In brief, the package was designed for BHF community facilitators to provide all of the training and materials necessary for program implementation. Specific training modules were developed for a program facilitator and for facilitators of the specific content areas (nutrition, physical activity, and lifestyle). Introductory modules also focused on program initiation and methods to improve program reach. Modules included presentation materials, handouts, lesson plans, facilitator knowledge checks, and self-assessed implementation fidelity checklists. To allow the program facilitator to track family progress and implementation fidelity a data portal was included in the package. The primary target of the BHF Online Training Resources and Program Package was to increase implementation fidelity (operationalized as adherence to protocol and increased facilitator capacity) while allowing adaptability of delivery within and across core intervention elements [26]. This strategy has secondary targets of improved adoption through reducing implementation complexity and improved reach.

### Building Health Families Action Learning Collaborative (BHF-LC) to improve implementation quality

The BHF-LC represents the primary independent variable for the HEI trial whereby 2 to 4 communities receive only the BHF Online Training Resources and Program Package (package only, BHF-PO) and 2 to 4 communities will receive the package and participate in a LC (BHF-LC). The intent of a LC is to bring together community teams with members from different organizations and professional roles (e.g., staff, supervisors, senior leaders) to work together to learn BHF and sustain its use over time. Activities used in the LC model include pre-work activities to build capacity, in-person trainings, and implementation periods between trainings that involve ongoing consultation and quality improvement strategies [6]. Specifically, the BHF-LC includes collaborative learning sessions followed by action periods over a 2-year implementation period. The learning session topics align with the timing of BHF program activities with an early focus on reach and recruitment, followed by a focus on implementation quality, followed by a focus on maintenance of community program implementation. Consultee-centered training [27] is embedded within learning sessions to improve facilitator implementation capacity. This includes instruction on core content, review of BHF implementation data, facilitator reflection on implementation processes, goal setting and feedback relative to implementation outcomes, and group-based problem-solving to improve reach, implementation fidelity, and sustainability. Finally, sustainability action planning will be included in the BHF-LC and focus on strategies to embed program implementation within organization workflows and job responsibilities. The sustainability action planning will also include engaging local payers and public health organizations to explore opportunities for ongoing funding and reimbursement for implementation.

### Evidence-based pediatric weight management intervention

BHF is a 12-month parent-child dyad PWMI program, adapted from Epstein and colleagues’ efficacious traffic light diet (TLD) PWMI, and was tailored to fit rural/micropolitan communities [20, 21]. The BHF program includes a minimum of 32 contact hours consisting of three main program components: nutrition education, behavior modification, and physical activity. Education is provided to children and parents together and independently based on the topic and depth of information [20, 21]. Participants and parents are expected to attend 12 continuous weeks of education (2 h/session) followed by 12 weeks of relapse prevention refresher courses (1 h per session for every 3 weeks). One additional refresher session (2 h) is conducted between 6 months and 1 year to revisit the TLD eating plan and environmental changes, re-ignite social support within the group, and finish with a fun activity. Two follow-up check-in sessions are conducted at 6 and 12 months for approximately 1 h per session.

### Eligibility, recruitment, and allocation

#### Community level

Applying the community engagement approach, the research team and the BHF-CAB disseminated the RFA within and outside their organizations and networks (i.e., applying network weaving strategy) and documented that communities across all 93 counties received the RFA. Eight communities with broad geographic distribution responded to the RFA of which seven rural communities in Nebraska completed a full proposal and were selected as the participating communities for the HEI trial. We then ranked the seven applications in a descending order and allocated the odd-numbered rankings to the (BHF-LC) and the even-numbered rankings to the package-only (BHF-PO) condition [23]. This exercise resulted in four communities in the BHF-LC condition and three communities in the BHF-PO condition. All communities that implement the PWMI will have access to the BHF Online Training Resources and Program Package that includes all of the materials necessary to implement and evaluate the BHF intervention.

Communities assigned to the BHF-PO condition are required to complete a memorandum of agreement (i.e., obtaining formal commitment strategy) to report on the implementation of the PWMI and to use pragmatic evaluation strategies for reach and effectiveness outcomes and to provide de-identified program data for baseline, 6, and 12 months. These communities will also have technical support as needed related to package function, but no other facilitation. Communities allocated to the BHF-LC condition will participate in LC training lessons with additional embedded implementation support activities with a goal to (1) develop local strategies to screen and engage families through the BMI Reporter in schools or clinical screening and referral, (2) increase implementation fidelity, and (3) plan for sustainability.

#### Individual level

Recruitment will be completed by selected communities through school BMI report cards, physician referral, local advertisements, and through word-of-mouth based on locally available resources and guided by the BHF Online Training Resources and Program Package. All materials are designed for local adaptation based on location, time of the session, clinical and school partners, and form of recruitment used. For BHF participation, families will be eligible if they have a 6- to 12-year-old child with a BMI percentile ranking at or above the 95th percentile. Children with major cognitive or physical impairments, parents or children with a contraindication for physical activity and families participating in a concurrent PWMI will be excluded. According to our previous studies [20–22], 8–12 families per cohort would be ideal with a minimum of 5 families and a maximum of 12 families in each cohort. The study goal is to enroll 12 families (*n*=30–48 individuals) per cohort and 2 cohorts per community. With a total of 7 communities recruited, the total PWMI intervention participants are approximately 420–672 parent-child dyads.

### Overview of economic evaluation

This protocol has been reported in line with the Consolidated Health Economic Evaluation Reporting Standards (CHEERS) checklist [28] (see Supplementary File 1: CHEERS Checklist).

The economic evaluation of the HEI trial is two-fold. First is to conduct a cost and cost-effectiveness analysis of the fund and contract dissemination strategy for the BHF adoption, the BHF Online Training Resources and Program Package relative to BHF adoption and implementation, and the BHF-LC implementation strategy for promoting BHF adoption, implementation fidelity, and sustainability for the duration of the trial (12 months). The second is to conduct a cost and cost-effectiveness analysis of the BHF intervention. The CEA will be conducted from an implementing community [29] and limited societal perspective (i.e., including only productivity loss of participants due to the participation in the BHF program). The perspective of implementing community would include costs of time spent on planning, training, recruitment, delivering, and post-session follow-up, resources used, communication with the research team and BHF participants, and opportunity costs of community facilitators. Moreover, we will include the time and costs of the research team for providing training, facilitation, and technical assistance. Costs associated with research administration, data collection, and trial outcome assessment will be excluded. All costs will be categorized as labor and non-labor costs (e.g., space, supplies, or information technology) and expressed as US $2022. The unit of analysis for the economic evaluation is the 7 communities consisting of approximately 21–28 community facilitators who will be provided with BHF Online Training Resources and Program Package with or without a LC implementation strategy to implement and deliver the BHF intervention. The time horizon of the within-trial CEA will be across a 15-month period of time (first 3 months for preparation, and later 12 months for BHF intervention), to incorporate sufficient time to complete the whole implementation process of one BHF cohort, initiating from preparation, training, recruitment, and program delivery to post-intervention follow-up. This differs from the primary clinical effectiveness time horizon of 6 months. Costs and benefits will not be discounted due to a short duration.

### Effect measurement

The outcomes measured across implementation strategies and BHF intervention are described in Table 1. At the community level, the outcome associated with the fund and contract dissemination strategy is BHF program adoption, defined as (1) an objective count of communities responding to call for LOI and full application and (2) an objective count of communities responding to RFA and contracted to implement BHF. In addition, the BHF Online Training Resources and Program Package strategy targets outcomes of adoption, measured as an objective count of numbers of BHF cohorts initiated, and implementation, operationalized as community facilitator competence using the knowledge check score obtained from the BHF online training resource and direct observation of implementation fidelity (i.e., adherence to protocol, engagement, and dose [i.e., the number and duration of sessions conducted per cohort]). Finally, the LC implementation strategy involves outcomes of (1) reach, measured as the number, proportion, and representativeness of eligible family screened; (2) adoption, measured as an objective count of BHF cohorts initiated; (3) implementation fidelity, measured using the fidelity checklist via direct observation conducted by the trained research staff; and (4) sustainability, operationalized as an objective count of BHF cohort continued following the completion of LC sessions.

At the individual level, the effectiveness outcome will be assessed using a matched cohort design to determine changes in BMI *z*-scores from baseline to 6 and 12 months among BHF intervention participants across BHF-LC and BHF-PO conditions. We will apply an intention-to-treat approach when conducting the economic evaluation of the BHF intervention (effectiveness as the outcome measure).

### Cost measurement

#### Labor costs

##### Dissemination and implementation strategies

The cost estimation of D&I strategies is guidied by the activity-based costing and Proctor framework, which is similar to the modified time-driven activity-based costing (TDABC) proposed by Cidav and colleagues [30]. We further extend the activity-based costing approach of implementation strategies to include opportunity costs, which are often overlooked and are key elements when considering the perspective of small-scale stakeholders [29], incurred by individuals (in our case, community facilitators) who are the target of D&I strategies. As the costs of D&I strategies are typically labeled as either labor (from performing the implementation activities) or non-labor costs (derived from the invoice or administrative data), the labor costs in a D&I strategy will be calculated by multiplying the hourly wages of personnel (*actor*) who conduct activities (*action*) associated with a D&I strategy with the amount of times and frequencies (*dose* and *temporality*) spent on activities plus the opportunity costs incurred by the strategy recipients (*action target*) (Table 2). The costs per D&I strategy will be calcuated as: ${C}_i={S}_i+\sum_{j=1}^m{X}_{ij}$, where *C*_*i*_ = costs per discrete D&I strategy *i*; *X*_ij_=costs associated with activity *j* assigned to a D&I strategy *i*; *j* = activity *j* associated with a D&I strategy *i*; *j* = 1, 2, 3, …, *m*; and *i* =1,2,3,...; and *S*_*i*_ = amount of non-labor costs assigned to implementation strategy *i*. We will use the national hourly wage rate, corresponding to the position identified, of each community facilitator, CAB members, and research team members for estimates of labor costs.

Table 2 details the specification of each D&I strategy used in the HEI trial and the application of the specification for estimates of labor costs. For example, categorized by implementation activities, the actor in the fund and contract dissemination strategy includes research team members, CAB members, and a project coordinator. The hourly wage rate of each personnel involved in a specific activity can be easily derived from the the US Bureau of Labor Statistics [31]. The activities involved in this strategy including drafting the LOI and the full applications to be distributed to interested communities that may respond to the RFA, and reviewing the application materials, meetings to discuss the application and make a decision, and contacting the awardees. To obtain the total cost for each implementation activity, we will multiply the total time spent on each activity by each research team and CAB member and community facilitator by their corresponding hourly wage rate. Similar exercises will be applied to all other activities. The total labor cost of a discrete D&I strategy will be derived by summing up the total cost of each implementation activity associated with that discrete strategy. Similarly, we will calculate the total labor cost of the LC strategy and the BHF Online Training Resources and Program Package by summing up the costs associated with each activity specified (Table 2), applying the similar steps stated above.

##### BHF program training, recruitment, and delivery

The cost of the BHF program is estimated by the following components—BHF online module training, participant recruitment, and BHF program delivery. We will use the similar costing approach proposed for D&I strategies to estimate the costs of BHF program training, recruitment, and delivery. The labor cost of the BHF program training, recruitment, and delivery will be estimated by the time spent on activities of training, participant recruitment, session preparation, delivery, and post-session follow-up and national hourly wage rates of the similar position of community facilitators who enacted the activities. In the limited societal perspective, we will take into account the opportunity cost of the families attended the intervention sessions and other use (e.g., individual consultation), using the average national average hourly wage rate [31], and the transportation costs, estimated by the time spent on transportation and standard mileage reimbursement [32]. Table 3 presents detailed cost specification of BHF program online training and delivery.

**Table 3:** Labor activity specification of implementing BHF intervention in the HEI trial by the Proctor framework [24]

Intervention	Outcome	Action	Actor	Target	Temporality	Dose (frequency)	Dose (duration)^**a**^
BHF program training, recruitment, & delivery	**Effectiveness** -BMI *z*-scores at baseline, 6-, & 12-month (child) -Weight/BMI (lbs.) at baseline, 6-, & 12-month (parents)	**BHF program delivery** -Information meeting -12 core courses across 12 continuous weeks -3 relapse prevention refresh courses -6-month assessment -1 relapse prevention refresh course -12-month assessment	-BHF-LC -BHF-PO	Child and parents dyads	-Baseline -First 12 weeks -Every 3 weeks -at 6 months -between 6 months & 1 year -at 12 months	Minimum 32 contact hours	-1 h -2 h per sessions -1 h per course -1 h -2 h -1 h
		**BHF program online training** -Information meeting -12 core courses across 12 continuous weeks -3 relapse prevention refresh courses -6-month assessment -1 relapse prevention refresh course -12-month assessment	-BHF-LC -BHF-PO	Child and parents dyads	Date of the training sessions	18 modules	Duration of the training sessions
		Recruitment/meeting/consultation	Research team	BHF-LC BHF-PO	Date of the consultations	As needed	Duration of the consultations

*BHF* Building Healthy Families, *LC* learning collaborative, *PO* Package only, *BMI* body mass index

^a^Preparation time and travel time (if any), and post-session follow-up (e.g., summary note) are also included

##### Non-labor costs

Non-labor costs include gym and classroom space, scales, diet books, printouts, handouts, educational supplies, equipment, and financial incentives provided for BHF participants and will be collected based on actual amounts spent and tracked from receipts and paid invoices. Costs of computers, Internet, and landline will not be included given that they are provided to community facilitators to conduct their daily tasks. To supplement the cost estimate of D&I strategies, we will also obtain cost information during the exit interview of community facilitators, as suggested by the literature [14].

##### Data collection

To track all the activities associated with a D&I strategy and BHF program online training, recruitment and delivery, we use a publicly available time-tracking tool (i.e., Clockify), which is accessible through the website or smartphone app, to monitor the activities logging in by the community facilitators or the research team. To mitigate the potential respondent burden, we pre-populate the identified implementation activities associated with each D&I strategy proposed in the trial on the Clockify platform. Community facilitators in both conditions will be asked to record their BHF associated activities if any (typically the day before and the day of the BHF sessions) using either the web- or mobile-based Clockify app. They will receive weekly reminders for weekly implementation activity tracking, regardless of their group assignment, through an automated system email sent out during the weekend. In addition, the project coordinator will engage with the communities for reminders via emails, text messages, or phone calls if any given data was not logged in the system. We will supplement the cost data collection with meeting attendance sheets and minutes (for the research team and CAB members) and a BHF session check-in sheet (for participants).

##### Data analysis

Table 1 summarizes the proposed within-trial economic evaluation and associated costs and outcomes by the RE-AIM indicators. The cost-effectiveness of D&I strategies is assessed by combing their associated costs and outcomes using an incremental implementation cost-effectiveness ratio [33] (ICER) if a strategy is simply applied to the BHF-LC with the BHF-PO as the comparison group or average implementation cost-effectiveness ratio [34] (ACER) if a D&I strategy is applied in both the BHF-LC and BHF-PO conditions (and thus no comparison group). The interpretation is similar to the interpretation for a trationdtional ICER. An ICER is interpreted as the extra cost incurred for the intervention to achieve an extra 1 unit of increase in outcomes (reach, adoption, implementation, and maintenance) measured, whereas ACER represents the cost associated with a one-unit change on given measures for the outcome of interest. For example, the ACER for the fund and contract dissemination strategy to increase the adoption of the BHF program, at the micropolitan and surrounding rural areas is described as the costs associated with one community responding to RFA and contracted to implement BHF.

Similarly, the cost-effectiveness of the BHF program is determined by the total LC costs (BHF-LC group only) and the BHF program costs consisting of training, recruitment, program delivery, and post-follow-up, and the effect of BMI *z*-score reduction at 6 and 12 months. The ICER result is interpreted as the cost for an additional unit decrease in BMI *z*-scores between the BHF-LC and BHF-PO conditions.

To ascertain whether a given ICER/ACER is cost-effective, we will compare them to thresholds that are based on findings from previous economic research [13].

##### Sensitivity analysis

Performing a sensitivity analysis is a critical component of an economic evaluation when the assumptions and parameter estimates applied in the analysis have uncertain precision. Two approaches will be employed in the proposed economic evaluation. The first approach is one-way deterministic sensitivity analysis (DSA) [35] for the cost analysis of D&I strategies and BHF program training, recruitment and delivery. We will conduct the DSA around key unit costs, cost components, and outcome measures (i.e., BMI *z*-score) to assess the impact of these changes/variations on the total costs and cost-effectiveness [36, 37]. We will use tornado diagrams to summarize the effects of varying key input parameters one at a time on the results of the ICER/ACER [38]. Parameters to be considered include but are not limited to the role and salary level of key personnel, intensity/number of BHF sessions, and changes in the lengths of attendance time for the session. The parameters will be sorted in descending order by their influence on the ICER/ACER. The longer bars indicate the most important parameters. The second approach is a nonparametric bootstrap simulation, and it will be used to construct the joint uncertainty (e.g., 95% confidence interval) for the incremental costs and incremental effects (i.e., BMI *z*-score reduction) between BHF-LC and BHF-PO conditions. In the bootstrap simulation, 2000 random samples of cost-effect pairs will be selected with replacement from the original trial dataset. Simulation results will be presented graphically using a scatter plot (i.e., the incremental cost-effectiveness plane [ICEP]) [39], where each dot represents the ICER of one iteration of the bootstrap simulation. In addition, we will conduct CEA of BHF intervention using parents’ weight loss outcomes at 6 and 12 months. Moreover, we will conduct scenario analyses under different settings and conditions to estimate replication costs to enhance decision-making for potential adopting communities [40, 41].

All analyses will be performed using Stata 16.0 (StataCorp LP, College Station, TX) or Microsoft Excel.

## Discussion

This protocol sets out our plans to assess the cost and cost-effectiveness of D&I strategies, utilized in a type III HEI trial to promote the adoption of an evidence-based PWMI (i.e., BHF) via a trial-based economic evaluation. Through this publication, we aim to inform the research and public health communities of the potential cost and benefits of implementing this type of work. Moreover, we will provide a case study of an economic plan by the RE-AIM indicators.

Economic consideration is often critical to decision makers who have to decide about the cost of adopting, implementing, and sustaining evidence-based interventions (EBIs) [42] as well as the costs related to D&I strategies applied to increase the rate of adoption, quality of implementation, and the likelihood of sustainability. Powell and colleagues [17] identified the need to increase economic evaluations of D&I strategies as one of five research priorities for advancing D&I science. Current areas of challenge in this pursuit include a lack of distinction between strategies that would promote adoption of an EBI with strategies that would promote high-quality implementation, and strategies that would improve the likelihood of sustainability, as well as the lack of a pragmatic and generalizable approach for gathering information on strategy costs, capturing adaptations and changes in strategies made during implementation, and the conduct of implementation economic evaluation.

These stem from a fundamental difference between implementation economic evaluation and traditional economic evaluation. The former addresses the resources (e.g., implementation activities) needed to encourage adoption, implementation, and sustainability of the EBI, whereas the latter focuses primarily on the start-up and ongoing costs of the EBI itself [43]. D&I strategies can vary in their intensity and resource use as well as their effectiveness in aiding the implementation of EBIs [6]. In this economic evaluation protocol targeting D&I strategies, we attempt to identify and present the strategy costs across RE-AIM dimensions to clarify sometimes fuzzy boundaries between these strategies. Moreover, to conduct cost assessment across RE-AIM dimensions, it may be implicitly assumed that one implementation strategy has an impact on only one implementation outcome, which is likely not the case in the present study. For example, the strategy of “developing packaged implementation blueprint” has impacts on three implementation outcomes (reach, adoption, and implementation). Even a fund and contract dissemination strategy with a primary goal to increase adoption embedded the need for network weaving to improve implementation and likely sustainability over the life course of the program. To not overestimate the cost-effectiveness (i.e., double-counting) of a D&I strategy, we deliberately attributed the activities within the strategy to an associated primary implementation outcome target for the given strategy.

Furthermore, we conduct CEA using costs related to each implementation outcome to demonstrate the potential to separate the strategy costs and present the CEA results by RE-AIM outcomes. Most importantly, we follow labels and definitions for the implementation strategy components based on the Expert Recommendations for Implementing Change (ERIC) taxonomies [6], which is commenced to address the challenges and difficulties of reconciling the terms and definitions of strategies, to allow for comparisons of our work to other studies examining the utility of different implementation strategies. However, we also recognize the challenge that the application of ERIC taxonomies is less than optimal in the real-world settings, especially in the community context—no clear cut between the discrete ERIC strategies and also the combinations of ERIC strategies and non-ERIC strategies. Moreover, the strategies used may change over the life course of intervention. It is critical to systematically document the adaptation made during the implementation of the programs [44], using the well-known Stirman framework [45], the LISTS approach proposed by Smith et al. [46, 47], or the FRAME-IS approach [48], and record the costs associated with adaptation activities along the course of the intervention.

Identification of costs incurred when adopting, implementing, and delivering an EBI is key to addressing the dissemination and implementation of EBIs and the strategies used to close the research-practice gap [49–52]. Unfortunately, costs are infrequently collected or reported with limited quality (mainly due to a non-standardized approach) and scope for understanding the costs or cost-effectiveness of implementation strategies [53–55]. There is an urgent need to provide a pragmatic and replicable methodology for costing D&I strategies to help stakeholders or community organizations decide what strategies are most efficient at achieving the goal of adoption, implementation, and sustainability. To facilitate the cost reporting of D&I strategies, several efforts have been made to the costing methods and applications [14, 30, 56–58]. In two recent studies, for example, Jordan et al. [56] reported using electronic cost capture methods to track personnel time associated with implementation preparation activities of a pediatric obesity intervention in primary care. Ritchie et al. [57] estimated the time and organization costs of facilitating the implementation of mental health integration in primary care with a structured spreadsheet including pre-specified facilitation activities, duration, and participating personnel. Although their approach may provide an accounting of resources for a particular setting, these results may not accurately generalize to other settings or be comparable since they simply reported partial costs in a subset of activities rather than across the entire phases of the implementation (pre-implementation costs in Jordan et al. [56] and implementation costs in Ritchie et al. [57]).

Notably, Cidav and colleagues [30] presented a pragmatic costing method integrating TDABC and the Proctor framework for the identification, specification, and reporting of implementation strategies [24]. However, the three parameters (frequency of the activity, average time to perform the activity, and unit costs of the resources to perform the activity) included in the TDABC may not appropriately capture costs or apply to implementation strategies due to intertwined elements and activities incorporated in each discrete D&I strategy. Furthermore, they did not account for the opportunity costs incurred by the action target (individuals who received the intervention) in the cost estimate formula of implementation strategies. Capturing implementation strategies can be challenging, as responsibility for implementation is often diffuse and strategies may be flexibly applied as barriers and challenges emerge [59]. Our proposed pragmatic and generalizable costing approach for D&I strategies with modified activity-based costing integrated with the Proctor framework applying a publicly available software will facilitate the cost estimate and tracking ability of all participating communities. The results will also demonstrate the usability of the proposed time-tracking tool for implementation activities to facilitate cost assessments of D&I strategies in the community settings, which holds the potential to be further disseminated to community-based practice/organizations with limited capacity or resources for cos tracking. Furthermore, the replication costs derived from the scenario analysis may shed some light on addressing the economic consideration of research translation by providing informed decision making for stakeholders who are interested in adopting the program with the aforementioned D&I strategies in their communities [41].

### Challenges and limitations

To our knowledge, this is one of few studies to conduct the economic evaluation by distinguishing among strategies that would promote adoption of an EBI from those that would promote high-quality implementation and from those that would improve the likelihood of sustainability. While we are setting out the venue to distinguish and collect the data to establish the economic case of implementation and D&I strategies in a type III HEI trial, there are a number of anticipated challenges of the work and associated potential limitations. First, it is of great possibility that community facilitators may not constantly record their implementation activities regardless of the ease of use of digital-based time tracking tools and the regular reminders sent out by the research team and the time tracking system. To remedy this concern, the documented activities will be complemented by the information gathered through research team field notes and from exit interviews, and also the activity log tracked by the research team for a robust cost assessment of D&I strategies. Second, the HEI trial is being conducted in the micropolitan and surrounding rural communities of Nebraska, where system and population characteristics may vary across different counties/communities. The proposed costing approach and economic evaluation framework may not be applicable to other non-micropolitan settings. To improve the replicability, we will explore the potential of the proposed economic evaluation in other settings, populations, and implementation strategies in an attempt to detangle the complexity of the work as described above and also the results under replication costs. The goal of this pilot economic evaluation is focusing more scientifically on teasing out costs by specific D&I strategies related to specific D&I outcomes. As such, our study does not address the use of these cost evaluations for decision makers, though we hypothesize that by engaging our CAB and community implementation teams on reviewing our findings that we will gain insight on how better to communicate cost evaluation as a decision aid in communities considering PWMI implementation. Finally, due to the limited sample size of the study population of this protocol (*n*=21–28 community facilitators), it may be underpowered to detect the cost-effectiveness of a LC implementation strategy plus BHF Online Training Resources and Program Package (BHF-LC) compared to BHF Online Training Resources and Program Package access only (BHF-PO) on the adoption, implementation, and sustainability of implementing the BHF program.

## Conclusion

The proposed costing approach and results of the economic evaluation of D&I strategies and implementing an EBI will contribute to the evolving but still scant literature on costing implementation strategies (and especially those for adoption and sustainment) and fill the evidence gap on the cost-effectiveness of D&I strategies. The results should assist policy makers, community organizations, local health departments, and stakeholders in potential adopting settings for an informed decision making regarding the sustainability and future adoption and dissemination.

## Supplementary Information

**Additional file 1.** CHEERS Checklist.

## Data Availability

There are no datasets associated with this protocol.

## Abbreviations

EBIs: Evidence-based interventions
BHF: Building Health Families
CEA: Cost-effectiveness analysis
HEI: Hybrid effectiveness-implementation trial
LC: Learning collaborative
RE-AIM: Reach, effectiveness, adoption, implementation, and maintenance
PWMIs: Pediatric weight management interventions
D&I: Dissemination and implementation
CAB: Community advisory board
RFA: Request for applications
TLD: Traffic light diet
BMI: Body mass index
TDABC: Time-driven activity-based costing
LOI: Letter of intent
ICER: Incremental implementation cost-effectiveness ratio
ACER: Average implementation cost-effectiveness ratio
DSA: Deterministic sensitivity analysis
ICEP: Incremental cost-effectiveness plane
ERIC: Expert Recommendations for Implementing Change
FRAME-IS: Framework for Reporting Adaptations and Modifications to Evidence-based Implementation Strategies
LISTS: Longitudinal Implementation Strategies Tracking System
